# Effect of Dietary Protein and Lipid Level on Growth, Antioxidant, and Gene Expression of Juvenile *Parabramis pekinensis*

**DOI:** 10.1155/anu/9923321

**Published:** 2025-05-15

**Authors:** Wentao Xu, Ye Xu, Zhijing Yang, Yaming Feng, Huanhuan Huo, Xiaoping Miao, Hailong Gu

**Affiliations:** ^1^Taizhou Institute of Agricultural Science, Jiangsu Academy of Agricultural Sciences, Taizhou 225300, China; ^2^Animal Science and Technology College, Jiangxi Agricultural University, Nanchang 330000, China; ^3^Jiangzhiyuan Fishery Technology Co., Jingjiang, Taizhou 225300, China

**Keywords:** antioxidant, growth, *Parabramis pekinensis*, Nrf2 pathway, target of rapamycin (TOR) pathway

## Abstract

Unreasonable ratio of protein to lipid in feeds could affect growth, antioxidant, and related pathway genes expression. This study aimed to investigate the suitable proportion of protein to lipid in feed with *Parabramis pekinensis*. The ratio protein-lipid (*P*/*L)* indicated by G1 (2.52), G2 (3.16), G3 (4.03), G4 (5.33), G5 (7.49), and G6 (11.67), which were fed to *P. pekinensis* (80 ± 10.52 g) for 56 days. The present results showed that diets with a protein-to-lipid ratio of approximately 3.5:1 (35% protein and 10% lipid, or less) were optimal for enhancing growth parameters, including body weight, WGR, PER, VSI, HSI, SGR, and feed conversion ratio (FCR). The quadratic regression analysis of FCR and protein efficiency ratio (PER) in *P. pekinensis* showed that *P/L* ratio performed best around 5.33. As the *P/L* ratio in feeds turned down, the best growth performance appeared at about 5.33 (*p* < 0.05), which was due to the unbalanced feed protein and fat levels. Meanwhile, *P/L* in 5.33 group exerted a protective function against oxidative damage in *P. pekinensis*. In addition, the increased antioxidant capacity contributed to the growth performance of the fish in 5.33 group, which showed the connection obviously. Thus, the connection existed in target of rapamycin (TOR) and Nrf2 signaling pathway, which was downregulated when the *P/L* ratio was around 2.52 and 11.67. On the contrary, the *P/L* ratio around 5.33 could enhance the expression of *tor* and *s6k1* to improve the growth of *P. pekinensis*. In the Nrf2 signaling pathway, the expression of *keap1*, *sod1*, and *gpx* affected antioxidant ability and the *P/L* ratio from 4.03 to 7.49 could be able to balance the antioxidant capacity, maintaining in normal level of *P. pekinensis*.

## 1. Introduction

As a commercially valuable freshwater species in East Asia, *Parabramis pekinensis* holds significant cultural and economic importance in Chinese aquaculture. While intensive breeding programs have successfully restored wild populations and achieved fifth-generation artificial propagation with improved growth performance [1]. Critical knowledge gaps persist in nutritional optimization, particularly regarding protein-lipid (*P*/*L*) ratios in formulated feeds. Addressing this deficiency is essential to sustain breeding profitability while minimizing environmental impacts.

Protein serves as the fundamental nutrient for fish growth and immune function. However, excessive inclusion could elevate feed costs and exacerbate nitrogenous waste discharge [2, 3]. Therefore, utilizing nonprotein energy to save protein has become a hot topic in fish nutrition and feed science [4, 5]. Considering this, much research has been conducted to decrease dietary protein incorporation by increasing the lipid content in fish diets [6]. As a result, it is crucial to provide correct *P/L* ratio feed during the culture process after breeding. Fish have a high utilization capacity of lipid, with which the utilization factor reaches more than 90% in growth, decomposition, and supplying energy. Lipid have high-calorie properties in feed, which production is higher than sugar and protein. Furthermore, lipid accumulates in body, forming fat, which is broken down for energy when the body needs. Therefore, feed lipid reduces protein consumption to save feed protein amount. Many reports have shown that fish employ lipid to save protein [7–9]. *P/L* ratio is an important index of feed design, which affects fish growth, body composition, antioxidant, and related genes expression. Meanwhile, the feeds could affect the antioxidant system of liver, which is used as a criterion for evaluating reactive oxygen species (ROS) in organisms [10].

Amino acids in fish can activate the target of rapamycin (TOR), which is used to regulate cell growth and metabolism (TOR) [11, 12]. In fact, the TOR signaling pathway regulates relevant factors to take part in the metabolism in cells, which play a crucial role in regulating protein synthesis in fish [13]. Recent advances highlight the dual regulatory roles of *P*/*L* ratios in nutrient metabolism. Mechanistically, dietary amino acids activate mTOR signaling to orchestrate protein synthesis [14], whereas lipid peroxidation derivatives modulate Nrf2-mediated antioxidant responses [15]. This metabolic crosstalk necessitates systematic evaluation of *P*/*L* impacts beyond conventional growth metrics. Current feed formulations for *P. pekinensis* remain empirically derived, lacking molecular-level validation of nutrient utilization efficiency. In the present study, we designed 6 feeds with different *P/L* ratios. It is necessary to know which *P/L* ratio feeds are associated with changes in TOR and Nrf2 signaling pathways. Figuring out the *P/L* ratio in feeds helps to achieve efficient feed utilization and is essential for maintaining optimal growth of *P. pekinensis*.

## 2. Materials and Methods

### 2.1. Diet and Fish Preparation

We designed 6 diets with different protein to lipid ratios, including G1:2.52 (P30.6/L12.1); G2:3.16 (P32.9/L10.4); G3:4.03 (P35.1/L8.7); G4:5.33 (P37.3/L7); G5:7.49 (P39.5/L5.3); G6:11.67 (P41.6/L3.6), respectively. Diets were air-dried at 25 °C for 24 h and then stored at −20 °C until used. Formulation is presented in Table 1. The juvenile *P. pekinensis* were obtained from Binjiang Aquaculture Breeding Farm in Jingjiang City, Jiangsu Province and then acclimated for 2 weeks in indoor concrete tanks (3.0m × 3.0m × 2.0 m, length × width × depth). After the acclimatization, 540 juvenile *P. pekinensis* (80 ± 10.52 g) were randomly assigned to 18 tanks (same as above), and each group had three replications (30 fish per pool). Fish were hand-fed to apparent satiation twice daily (8:30 and 16:30) for 8 weeks. We exchanged the water every 7 days. During culturing, water temperature, dissolved oxygen level, and ammonia nitrogen level were 26.0 ± 0.8 °C, 7.0 ± 1.2 mg·L^−1^, and 0.15 ± 0.05 mg·L^−1^, respectively.

### 2.2. Sample Collecting

After 56 days of culturing, the fish were all fasted for 24 h and were anesthetized with 100 mg·L^−1^ MS-222 for sampling and acquiring the weight and number of fish. Nine fish per tank were randomly collected to use, in which three fish were stored at −20 °C for body composition analysis, and the other fish collected livers and intestines were stored at −80 °C until used.

### 2.3. Analysis

#### 2.3.1. Proximate Composition

We dried the fish to a constant weight in an oven at 105 °C to measure moisture content and burned the sample in muffle furnace at 550 °C to measure ash content. The Kjeldahl nitrogen method (*N* × 6.25) (Kjeltec 2200, FOSS, Denmark) was used to measure crude protein, and the Soxhlet method (Soxhlet extraction system B-811) by petroleum ether extraction was used to determine crude lipid content.

#### 2.3.2. Antioxidant Enzyme Activity

Liver and intestinal tissue were diluted with 10 times of 0.86% saline (ice water bath). Then we centrifuged tissues at 8000 r/min for 10 min at 4 °C and collected the supernatant for the relevant biochemical parameter measurements. The liver nutritional metabolism like alkaline phosphatase (ALP, No. A059-2-2) activities, aspartate aminotransferase (AST, No. C010-2-1), lipase assay kit (LPS, A054-2-1) contents and antioxidant capacity like superoxide dismutase (SOD, No. A001-3-2), catalase (CAT, No. A007-1-1), and total antioxidant capacity (T-AOC, No. A015-2-1), reduced glutathione (GSH, No. A006-2-1) and malondialdehyde (MDA, No. A003-1-2) contents were measured using commercially available kits (Nanjing Jiancheng Bioengineering Institute, Nanjing, China), as well as the gut *α*-amylase (AMS, No. C016-1-2) contents.

#### 2.3.3. Growth-Related Genes Expression and Real-Time PCR Analysis

Total RNAs were isolated with TaKaRa MiniBEST Universal RNA Extraction Kit (Takara) following the manufacturer's instructions. RNA degradation and contamination were monitored on 1% agarose gels. The purity and concentration were checked using the Nanodrop 2000 spectrophotometer (Thermo Scientific, USA). The criteria used to select the RNA for subsequent analysis were OD260/280 ≥ 1.8, 28S/18S rRNA ratio ≥ 1.0, and the amount and concentration of total RNA ≥ 3 μg and 50 ng/μL, respectively. Reverse transcription was conducted based on 2.0 μg total RNA using PrimeScript first Strand cDNA Synthesis Kit (Takara) following the manufacturer's instructions.

qPCR was run using TB Green Fast qPCR Mix (TaKaRa) on a CFX96 real-time PCR detection system thermocycler (Bio-Rad). The specific primers used for qPCR are listed in Table 2. Nine RNA samples per group were used in qPCR analyses. Each sample was run in triplicate, and the average threshold cycle (Ct) was normalized using the geometric mean of ubiquitin/ribosomal S27 fusion protein (s27) gene. We calculated relative gene expression by the 2^−*ΔΔ*Ct^ method. Efficiency (*E*) of qRT-PCR was ranged from 90% to 110%. Primers of growth-related genes were listed in Table 2.

### 2.4. Calculations and Statistical Analysis

The parameters were calculated using the following formulas, which we used before [16]:

  $$\begin{array}{c} \text{Feed intake}\left(\%/\text{day}\right)=100\times \frac{\text{feed consumption}}{\left(W_{t}+W_{f}\right)/2\times d} \end{array}$$

  $$\begin{array}{c} \text{Weight gain rate }\left(\text{WGR},\%\right)=100\times \frac{W_{f}-W_{i}}{W_{i}} \end{array}$$

  $$\begin{array}{c} \text{Specific growth rate}\left(\text{SGR},\%/\text{day}\right)=100\times \frac{\text{In }w_{f}-\text{In}w_{i}}{d} \end{array}$$

  $$\begin{array}{c} \text{Feed conversion ratio }\left(\text{FCR}\right)=\frac{\text{feed consumption}}{W_{f}-W_{i}} \end{array}$$

  $$\begin{array}{c} \text{Protein effciency ratio }\left(\text{PER}\right)=100\times \frac{W_{f}-W_{i}}{\text{protein take}} \end{array}$$

  $$\begin{array}{c} \text{Conditionfactor}\left(\text{CF},g/{\text{cm}}^{3}\right)=100\times \frac{\text{body weight}\left(\text{g}\right)}{\text{body length}{\left(\text{cm}\right)}^{3}} \end{array}$$

  $$\begin{array}{c} \text{Viscerosomatic index}\left(\text{VSl},\%\right)=100\times \frac{\text{viscerosomatic weight}\left(\text{g}\right)}{\text{whole body weight}\left(\text{g}\right)} \end{array}$$

  $$\begin{array}{c} \text{Hepatosomatic index }\left(\text{HSI},\%\right)=100\times \frac{\text{hepatic weight}\left(\text{g}\right)}{\text{whole body weight}\left(\text{g}\right)}. \end{array}$$

We used SPSS 20.0 software to process the data and showed the results as the mean ± SD (standard deviation). All results were tested for normality and homogeneity of variances. One-way ANOVA and Duncan's multiple range test were used to analyze the differences among these results. The significance threshold was *p* < 0.05.

## 3. Results

### 3.1. Growth Performance and Proximate Composition

**Figure 1:**
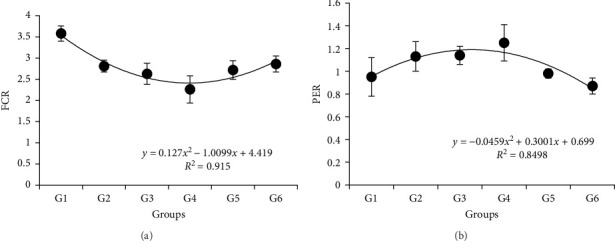
Quadratic regression analysis of feed conversion ratio (FCR) (A) and protein efficiency ratio (PER) (B) in *P. pekinensis* fed with different *P*/*L* ratio contents.

Results showed that different *P/L* feeds did not affect the survival rate (SR) (Table 3). Body weight, WGR, PER, VSI, and HSI showed similar trends, which were improved with the increasing *P/L* ratio to 5.33 and depressed thereafter. Specifically, it showed more significance than other groups *p* < 0.05. The peak value of VSI appeared in 4.03, which was significantly higher than 5.33 and other groups (*p* < 0.05). The SGR was significantly lower in 2.52 and 11.66 compared to other groups (*p* < 0.05). The feed conversion ratio (FCR) showed that the 5.33 group was significantly higher than the 2.52 group (*p* < 0.05), while no difference with other groups (*p* > 0.05). Based on the quadratic regression analysis, the vertex of the curve was between 4.03 and 5.33 both in FCR and PER (Figure 1). Dietary with different *P/L* significantly affected the crude lipid content in whole fish (*p* < 0.05), which fell down with the increasing *P/L* ratio from 2.52 to 11.66. There was no difference observed in the moisture, ash, and crude protein contents of whole fish across all the groups (*p* > 0.05) (Table 4).

### 3.2. Physiological Metabolism

As shown in Table 5, the AST contents generally decreased with the *P/L* ratio up to 4.03 and then increased; the 4.03 group showed significant difference lower than 5.33 and 7.49 groups, which also showed differences lower than 2.52, 3.16 and 11.66 groups (*p* < 0.05). The LPS contents showed a decreasing trend from 2.52 to 11.66 and 11.66 group showed significant difference with other groups (*p* < 0.05). The 3.16 group showed difference in AMS contents (*p* < 0.05), and other groups maintained at the same level.

### 3.3. Antioxidant Status

Different *P/L* ratio feeds had no remarkable impact on the activities or contents of T-AOC and CAT in liver (*p* > 0.05; Table 6). But the CAT contents suggested the same trend with the SOD contents, which had an increase trend from 2.52 to 7.49, while the trend reversed in 11.66. The 7.49 group had the highest level among them (*p* < 0.05), however, there was no difference between 7.49 and 5.33 groups (*p* > 0.05). Conversely, the MDA content generally decreased from 2.52 to 5.33 and then increased, which was significantly lower in 5.33 and 11.66 compared to other groups (*p* < 0.05). The GSH activity showed no difference from 2.52 to 4.03, and they were in an intermediate position. Interestingly, the 5.33 group got the minimum level, and 7.49, 11.66 groups got the highest level (*p* < 0.05).

### 3.4. Relative Gene Expression

**Figure 2:**
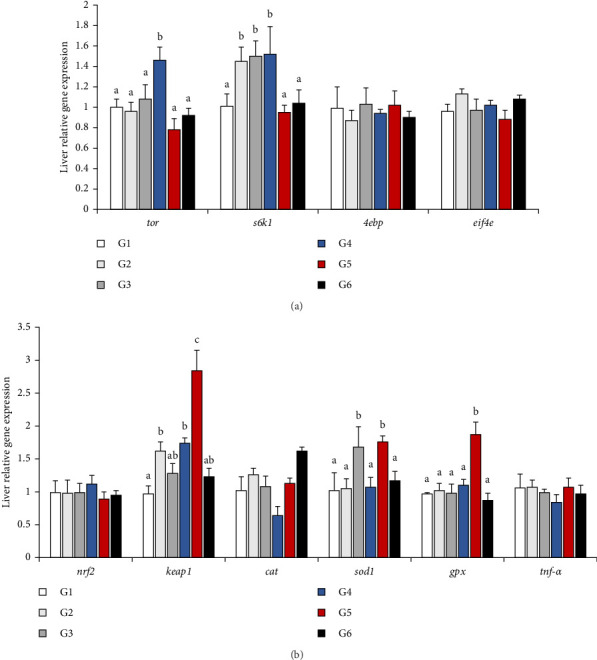
Relative gene expressions of *P. pekinensis* in liver. (A) TOR signaling pathway and (B) Nrf2 signaling pathway. Data are expressed as a graphical representation of the mean and error bars (means ± SEM). Mean values with different letters superscripted^abc^ above bars indicate the effect was significantly different between groups (*p* < 0.05).

The relative gene expression was depicted as a histogram in Figure 2. In the TOR signaling pathway, *4ebp* and *eif4e* expression did not significantly differ among all groups (*p* > 0.05, Figure 2A). Similarly, the expression levels of tor and *s6k1* showed the same trend, which appeared a mountain shape, with the changes of *P/L* ratio in feeds. The 5.33 was significantly higher than other groups in tor expression and it was significantly higher than 2.52, 7.49 and 11.66 in *s6k1* expression (*p* < 0.05). In the Nrf2 signaling pathway, *nrf2*, *cat*, and *tnf-α* did not significantly differ among all groups (*p* > 0.05, Figure 2B). However, the 7.49 group presented the highest expression levels of the genes related to the immune response and *keap1*, *sod1*, and *gpx* expression in the Nrf2 signaling pathway suggested the highest value (*p* < 0.05). It was worth mentioned that keap1 expression level in 3.16 was equal to 5.13, which were showed difference with 2.52 (*p* < 0.05), and sod1 expression level in 4.03 was equal to 7.49, which were showed difference with other groups (*p* < 0.05).

## 4. Discussion

The study of protein and lipid ratio in feeds was fundamental to the low-cost of development, balanced nutrition, and environment-friendly feeds [17]. Meanwhile, the protein-to-lipid (*P/L*) ratio in feed was a key factor affecting the growth performance of fish [18]. In this study, we found that different *P/L* feeds made no significant difference in the SR of juvenile *P. pekinensis*. Consistently, similar results were also found in juvenile rice field eel [19]. Our results in body weight, WGR, PER, VSI, HSI, SGR, and FCR showed G4 group (5.33 (P37.3/L7)) performed well and it suggested that diets formulated with 35% protein and no more than 10% lipids were recommended for optimizing growth. In fact, the *P/L* ratio requirement was verified in *Macrobrachium acanthurus*, *Carassius auratus* Var. Pengze and *Megalobrama amblycephala* [20–22]. The quadratic regression analysis of FCR and PER in *P. pekinensis* showed that *P/L* ratio performed best around 5.33. In the study, the optimum FCR value was about 2.26, which was higher than other freshwater carp [2, 23, 24]. It could be affected by indoor farming environment and demanding feed ingredients. The crude lipid contents were changed following the lipid contents in feeds, which were affected lipid-related materials. Except for crude lipid, no significant effects on the other body composition were observed in juvenile *P. pekinensis*, which was similar to the report on juvenile tilapia (*Oreochromis niloticus*) that fed on different carbohydrate and lipid levels feeds [25]. The crude lipid contents were changed following the feed lipid contents, which could be significantly changed with different diets. This result agrees with previous reports, which were consistent with the results of the hybrid grouper (*Epinephelus fuscoguttatus* × *Epinephelus polyphekadion*), *Rachycentron canadum*, and *Scophthalmus maximus L*. [26–28].

In the present study, different *P/L* ratio intake could make difference with VSI and HSI in fish [29, 30]. It was reported that AST and ALT were important to protein metabolism, glucose metabolism, and indicated liver function [31]; LPS and AMS played important roles in the process of lipid decomposition [32]. In this experiment, the best AST value appeared in the range of 4.03–7.49, and the LPS decreased with the change of lipid content. As the *P/L* ratio in feeds turned down, the best growth performance appeared at about 5.33, which was due to the unbalanced feed protein and fat levels. According to our analysis, *P. pekinensis* were intolerant to the unbalanced *P/L* ratio in feed, and nutrition balance in feed was the key factor. The conclusion was found in *S. maximus* [33] and *Solea senegalensis* [34]. In addition, other studies indicated that different types of lipids in feed had a great impact on digestion and utilization of nutrients in *Oncorhynchus mykiss* [35]. However, some researchers suggested that adding 10% vegetable oil, poultry oil, or marine fish oil to diet did not produce significant difference in *Micropterus salmoides* [36]. In summary, an unbalanced dietary *P/L* ratio, rather than fat source, was the key factor for the differences in growth performance of *P. pekinensis*.

As a vertebrate, *P. pekinensis* had enzyme-promoted and nonenzymatic antioxidant defense mechanisms, which could assist against oxidative stress [37]. Obviously, dietary was able to be manipulated and increased the antioxidant activity [38]. Meanwhile, the antioxidant system of liver played a key role in capturing and eliminating excess ROS. Previous studies showed that SOD, CAT, GSH, T-AOC, and MDA could be used as important indicators to of antioxidant capacity [39]. SOD activity was often used to assess the ability of scavenging superoxide anion radicals and protecting cells from oxygen radical damage [40]. And MDA was a metabolite produced by lipid peroxidation, and its level within the body was often used as a reference for the degree to which cells of the body are attacked by free radicals [41]. The stress of high-protein and high-lipid diet, which exceeds the antioxidant defense, could lead to many problems, such as hepatotoxicity and liver dysfunction, physiological and metabolic disorders, growth inhibition, and inflammatory responses Benitez-Hernández et al. [42]. SOD could catalyze the transformation of superoxide anion radicals into nontoxic compounds [43]. The extreme *P*/*L* ratio could cause damage to liver and the lipid metabolism capacity decreases with fat content. As the *P*/*L* ratio in feeds turned down, the best growth performance appeared at about 5.33, which was due to the unbalanced feed protein and fat levels. In the study, all G4 inclusion groups showed significantly increased of SOD and decreased of MDA, CAT (*p* < 0.05). All G3 and G5, which *P/L* ratio around G4, inclusion groups only showed difference in the enzyme index with G4, and other index were similar to G4. The above results showed that the inclusion of G4 exerted a protective function against oxidative damage in *P. pekinensis*. It was suggested that *P/L* in feeds only suitable to fish could operate well in antioxidants. In addition, it was interesting to note that the increased antioxidant capacity contributed to the growth performance of the fish in G4, which showed the connection obviously. Cho et al. [44] found that the protein requirement of rainbow trout (*Salmo gairdneri*) was 35% to 45%, and the lipid content could reach to 20% [45]. Under the right of protein and lipid, the fish growth and antioxidants could be promoted and produce synergy. The conclusion was found in juvenile hybrid grouper (*E. fuscoguttatus* × *E. lanceolatus*), juvenile shrimp (*Macrobrachium nipponense*), and juvenile cobia (*R. canadum*) [46–48].

The growth performance of *P. pekinensis* was positively correlated with *P/L* ratio. The specific value reflected the ability of protein synthesis [49], and stimulation of mTOR signaling pathway could enhance protein synthesis to promote growth in fish [50]. The liver not only absorbed and stored nutrients, but also served the metabolic center of the body [51]. The TOR was a serine/threonine protein kinase member of the phosphoinositide-3-kinase-related kinase family [52]. In this study, the expression levels of *tor* and *s6kl* involved in the TOR signaling pathway were upregulated first and then downregulated. However, the expression levels of *4ebp* and *eif4e* showed no difference. It suggested that *P/L* ratio of around 5.33 could activate TOR pathway, which is consistent with the study in *Portunus trituberculatus* [53]. Therefore, the reason for the different expression of TOR signaling pathway-related gene levels may be that the excessive or insufficient protein and lipid contents, which supply the nutrients to cells' growth and development. Furthermore, high-lipid diet could break down more fats into tissues and more free fatty acids are delivered to the liver, which leads to fibrosis and cirrhosis of liver and prolonged compression of body functions. Thus, the TOR signaling pathway was downregulated when *P/L* ratio was around 2.52 and 11.67. On the contrary, the *P/L* ratio of around 5.33 could enhance growth of *P. pekinensis*. Previous studies have revealed that nuclear factor erythroid 2-related factor 2 (Nrf2) could regulate antioxidant enzymes and corresponding genes to relieve oxidative stress [54, 55]. In this case, it protected cells from various injuries, thus influencing the course of disease. Our study suggested that *P/L* ratio around 7.49 showed more expression levels of *keap1*, *sod1* and *gpx*. And the *P/L* ratio around 4.03 showed more expression levels of *sod1*. Relevantly, the enzyme activities of SOD, CAT and GSH were affected by expression levels of Nrf2 pathway genes. It might be that *P/L* ratio in feeds more than 7.49 or less than 4.03 could produce an inflammatory response, which caused an increase in anti-inflammatory factors. Thus, the *P/L* ratio from 4.03 to 7.49 could be able to healthy body in *P. pekinensis*. Further research is needed to determine the exact mechanism.

## 5. Conclusions

In conclusion, our results suggested that diets with a protein-to-lipid ratio of approximately 3.5:1 (35% protein and 10% lipid, or less) were optimal for enhancing growth parameters. The quadratic regression analysis of FCR and PER in *P. pekinensis* showed that the *P/L* ratio performed best around 5.33. According to our analysis, *P. pekinensis* was intolerant to the unbalanced *P/L* ratio in feed, and nutrition balance in feed was the key factor. And the *P/L* ratio in feeds only suitable to fish that could operate well in antioxidants. In addition, the increased antioxidant capacity contributed to the growth performance of the fish in the 5.33 group, which showed the connection obviously. Thus, the connection existed in TOR and Nrf2 signaling pathway, which was downregulated when *P/L* ratio was around 2.52 and 11.67. On the contrary, the *P/L* ratio around 5.33 could enhance the expression of *tor* and *s6k1* to improve growth of *P. pekinensis*. In Nrf2 signaling pathway, the expression of *keap1*, *sod1* and *gpx* affected antioxidant ability and the *P/L* ratio from 4.03 to 7.49 could be able to balance the antioxidant capacity maintaining in a normal level of *P. pekinensis*. The *P/L* ratio around 5.33 showed the best performance in growth, antioxidant and gene expression.

## Figures and Tables

**Table 1:** Ingredient and proximate composition of experimental diets (dry matter %) .

Diet	G1	G2	G3	G4	G5	G6
Fish meal	10.00	10.00	10.00	10.00	10.00	10.00
Rapeseed meal	15.00	15.00	15.00	15.00	15.00	15.00
Cotton meal	20.00	25.00	30.00	35.00	40.00	45.00
Wheat flour	15.00	15.00	15.00	15.00	15.00	15.00
Soybean oil	5.00	4.00	3.00	2.00	1.00	0.00
Choline chloride	0.50	0.50	0.50	0.50	0.50	0.50
Vitamin C (35%)	0.05	0.05	0.05	0.05	0.05	0.05
Vitamin premix^a^	2.00	2.00	2.00	2.00	2.00	2.00
Ca (H_2_PO_4_)_2_	2.50	2.50	2.50	2.50	2.50	2.50
Mineral premix^b^	2.00	2.00	2.00	2.00	2.00	2.00
Rice bran	20.00	16.00	12.00	8.00	4.00	0.00
Microcrystalline cellulose	5.94	5.94	5.94	5.94	5.94	5.94
Ethoxy quinoline	0.01	0.01	0.01	0.01	0.01	0.01
Bentonite	2.00	2.00	2.00	2.00	2.00	2.00
Total	100.00	100.00	100.00	100.00	100.00	100.00
Crude protein (%)	30.6	32.9	35.1	37.3	39.5	41.6
Crude lipid (%)	12.1	10.4	8.7	7.0	5.3	3.6
Gross energy (KJ)	17.2	17.0	16.8	16.5	16.3	16.0
Digestible energy (KJ)	12.6	12.5	12.4	12.3	12.1	12.0
Lys	0.26	0.19	0.08	0	0	0
Met	0.31	0.36	0.38	0.40	0.44	0.46
Thr	0.15	0.14	0.08	0.03	0.01	0
Protein/Lipid (*P/L*)	2.52	3.16	4.03	5.33	7.49	11.66

*Note*: The diets commissioned from Jiangxi Agricultural University, including materials, premix and producing.

^a^Vitamins premix (IU, mg/kg of premix): Vitamin A, 900,000 IU; Vitamin D, 250,000 IU; Vitamin E, 4500 mg; Vitamin K3, 220 mg; Vitamin B1, 320 mg; Vitamin B2, 1090 mg; Vitamin B5, 2000 mg; Vitamin B6, 5000 mg; Vitamin B12, 116 mg; Pantothenate, 1000 mg; Folic acid, 165 mg; Choline, 60,000 mg; Biotin, 50 mg Niacin acid, 2500 mg.

^b^Mineral premix (g/kg of premix): calcium biphosphate, 20 g; sodium chloride, 2.6 g; potassium chloride, 5 g; magnesium sulphate, 2 g; ferrous sulphate, 0.9 g; zinc sulphate, 0.06 g; cupric sulphate, 0.02 g; manganese sulphate, 0.03 g; sodium selenate, 0.02 g; cobalt chloride, 0.05 g; potassium iodide, 0.004 g.

**Table 2:** The sequence of the primers used for real-time quantitative PCR.

Gene	Sequence 5–3′	Genbank accession no.
*tor*	F-GCAGCCCCAAGGAGATGAAA	XM_050855996
	R-ACAGTCGAAACGCCCTCATC	
*s6k1*	F-GCACCAGGCTTATTCGACCT	XM_050854928
	R-GGGTTGACTGTGCTGTCTGA	
*4ebp*	F-CACGAAACCGACTACTGC	XM_050856547
	R-CCAAGACCTGATGATGAAC	
*eif4e*	F-CAAGCAGTAGTCCCTCACAAA	XM_050881025
	R-GTGGCCTTACACACAGTAGTC	
*nrf2*	F-AGAGACATTCGCCGTAGA	NM_212855.2
	R-TCGCAGTAGAGCAATCCT	
*keap1*	F-CGTACGTCCAGGCCTTACTC	XP_018520553.1
	R-TGACGGAAATAACCCCCTGC	
*cat*	F-CTATGGCTCTCACACCTTC	MK_614708.1
	R-TCCTCTACTGGCAGATTCT	—
*sod1*	F-TGGCAAGAACAAGAACCACA	NC_007121.7
	R-CCTCTGATTTCTCCTGTCACC	—
*gpx*	F-GAAGGTGGATGTGAATGGA	MK_614713.1
	R-CCAACCAGGAACTTCTCAA	—
*s27*	F-GGTCGATGACAATGGCAAGA	XM_050861302
	R-CCACAGTACTGGCGGTCAAA	
*tnf-α*	F-CTTCGTCTACAGCCAGGCATCG	NC_048566.1
	R-TTTGGCACACCGACCTCACC	

**Table 3:** Descriptive statistic for growth performance indices (Mean ± SD; *n* = 9).


Diets	Final body weight (g)	Feed intake (%/day)	WGR (%)	SGR (%/day)	FCR	PER	CF (g cm^3^)	VSI (%)	HSI (%)	SR (%)
G1	145.85 ± 12.25^a^	2.52 ± 0.14	67.15 ± 7.56^a^	0.92 ± 0.04^a^	3.58 ± 0.18^b^	0.95 ± 0.17^a^	1.72 ± 0.04	8.73 ± 1.64^b^	2.39 ± 0.13^ab^	100.00 ± 0.00
G2	146.57 ± 10.25^a^	2.44 ± 0.16	89.75 ± 6.17^b^	1.14 ± 0.07^b^	2.81 ± 0.14^ab^	1.13 ± 0.13^b^	1.79 ± 0.02	9.24 ± 0.88^b^	2.19 ± 0.52^a^	96.67 ± 3.33
G3	170.92 ± 21.29^b^	2.39 ± 0.11	95.88 ± 8.73^b^	1.20 ± 0.03^b^	2.63 ± 0.25^ab^	1.14 ± 0.08^b^	1.78 ± 0.02	10.42 ± 1.50^d^	2.51 ± 0.34^b^	100.00 ± 0.00
G4	184.47 ± 14.91^c^	2.27 ± 0.23	111.41 ± 12.41^c^	1.34 ± 0.12^bc^	2.26 ± 0.32^a^	1.25 ± 0.16^c^	1.77 ± 0.07	9.89 ± 0.95^c^	2.50 ± 0.74^b^	100.00 ± 0.00
G5	168.29 ± 18.41^b^	2.41 ± 0.18	92.86 ± 10.36^b^	1.17 ± 0.09^b^	2.72 ± 0.22^ab^	0.98 ± 0.04^a^	1.85 ± 0.06	7.93 ± 1.54^a^	1.98 ± 0.78^a^	93.33 ± 6.67
G6	160.73 ± 11.02^b^	2.37 ± 0.15	84.21 ± 8.64^b^	1.09 ± 0.08^ab^	2.86 ± 0.19^ab^	0.87 ± 0.07^a^	2.20 ± 0.05	8.72 ± 0.75^b^	2.04 ± 0.48^a^	96.67 ± 3.33
*p* value	0.021	0.306	0.014	0.037	0.018	<0.001	0.058	<0.001	<0.001	0.549

Different letters denoted significant differences (*p* < 0.05).

**Table 4:** Descriptive statistic for proximate composition indices (Mean ± SD; *n* = 9).

Diets	Moisture (g/kg)	Ash (g/kg)	Crude protein (g/kg)	Crude lipid (g/kg)
G1	712.56 ± 23.12	23.23 ± 0.18	167.14 ± 5.75	18.79 ± 1.25^c^
G2	749.54 ± 20.87	24.95 ± 0.82	153.72 ± 4.22	17.84 ± 0.77^bc^
G3	753.86 ± 7.13	29.48 ± 0.24	160.28 ± 6.41	15.81 ± 0.82^b^
G4	742.65 ± 15.46	27.15 ± 1.45	162.54 ± 5.21	14.12 ± 1.48^b^
G5	741.83 ± 10.23	25.79 ± 0.66	161.54 ± 3.39	10.54 ± 0.26^a^
G6	740.45 ± 25.61	25.06 ± 1.82	157.64 ± 4.61	10.02 ± 1.69^a^
*p* value	0.784	0.825	0.878	0.034

Different letters denoted significant differences (*p* < 0.05).

**Table 5:** Descriptive statistic for liver biochemical and enzyme activities indices (Mean ± SD; *n* = 9).

Diets	AST (U g prot)	ALT (U g prot)	LPS (U g prot)	AMS (mg/min/g)
G1	629.74 ± 33.65^c^	709.25 ± 38.47	165.15 ± 10.47^c^	0.58 ± 0.07^b^
G2	572.45 ± 35.17^c^	832.19 ± 32.56	158.46 ± 5.98^c^	0.14 ± 0.02^a^
G3	314.15 ± 24.53^a^	744.84 ± 44.26	112.14 ± 8.67^ab^	0.37 ± 0.05^b^
G4	417.76 ± 20.68^b^	700.25 ± 32.69	106.28 ± 8.24^ab^	0.41 ± 0.03^b^
G5	450.77 ± 29.54^b^	688.79 ± 28.41	108.56 ± 6.41^ab^	0.43 ± 0.02^b^
G6	526.12 ± 22.78^c^	640.33 ± 37.98	88.15 ± 5.16^a^	0.46 ± 0.02^b^
*p* value	0.021	0.355	0.042	0.004

Different letters denoted significant differences (*p* < 0.05).

**Table 6:** Descriptive statistic for liver immunity enzyme activities indices (Mean ± SD; *n* = 9).

Diets	SOD (U mg prot)	MDA (nmol mg prot)	T-AOC (mmol g prot)	CAT (U/g)	GSH (μmol g prot)
G1	31.24 ± 3.12^a^	1.71 ± 0.21^b^	0.35 ± 0.08	952.18 ± 50.45	21.74 ± 2.17^b^
G2	37.75 ± 2.78^a^	1.67 ± 0.15^b^	0.27 ± 0.05	928.62 ± 46.87	20.27 ± 1.22^b^
G3	35.95 ± 3.56^a^	1.54 ± 0.08^b^	0.36 ± 0.04	1179.57 ± 55.16	18.26 ± 1.85^ab^
G4	40.17 ± 3.28^ab^	1.28 ± 0.11^a^	0.32 ± 0.07	1087.15 ± 64.74	15.21 ± 1.24^a^
G5	43.48 ± 5.74^b^	1.45 ± 0.14^ab^	0.34 ± 0.06	1108.81 ± 31.26	29.91 ± 2.31^c^
G6	33.81 ± 4.11^a^	1.17 ± 0.16^a^	0.28 ± 0.04	940.42 ± 55.93	39.12 ± 3.67^d^
*p* value	<0.001	0.022	0.468	0.328	0.017

Different letters denoted significant differences (*p* < 0.05).

## Data Availability

The data that support the findings of this study are available from the corresponding author upon reasonable request.
